# Cuttlefish retrieve whether they smelt or saw a previously encountered item

**DOI:** 10.1038/s41598-020-62335-x

**Published:** 2020-03-25

**Authors:** P. Billard, N. S. Clayton, C. Jozet-Alves

**Affiliations:** 1 Normandie Univ, Unicaen, CNRS, EthoS, 14000 Caen, France; 2 Univ Rennes, CNRS, EthoS (Éthologie animale et humaine) - UMR 6552, F-35000 Rennes, France; 3 Department of Psychology, University of Cambridge, Cambridge, CB2 3EB UK

**Keywords:** Animal behaviour, Learning and memory

## Abstract

According to the Source Monitoring Framework, the origin of a memory is remembered through the retrieval of specific features (*e.g*. perceptive, sensitive, affective signals). In two source discrimination tasks, we studied the ability of cuttlefish to remember the modality in which an item had been presented several hours ago. In Experiment 1, cuttlefish were able to retrieve the modality of presentation of a crab (visual vs olfactory) sensed before 1 h and 3 hrs delays. In Experiment 2, cuttlefish were trained to retrieve the modality of the presentation of fish, shrimp, and crabs. After training, cuttlefish performed the task with another item never encountered before (e.g. mussel). The cuttlefish successfully passed transfer tests with and without a delay of 3 hrs. This study is the first to show the ability to discriminate between two sensory modalities (*i.e*. see vs smell) in an animal. Taken together, these results suggest that cuttlefish can retrieve perceptual features of a previous event, namely whether they had seen or smelled an item.

## Introduction

Can you tell whether you *truly* enjoyed your last holiday? According to the Source-Monitoring Framework (SMF), answering such a question requires you to revisit your personal past and retrieve specific features belonging to your memories (*e.g*. affective, perceptual and contextual features^1^). For instance, I can remember that I went to my parents’ home town (contextual features), and that we spent evenings talking or playing music (perceptual features) in a joyful atmosphere (affective feature). To remember these specific details and moments, I travelled mentally back through my personal past and engaged in *episodic cognition processes*, projecting myself in space and time to re-live and re-experience the content of those personal memories, integrating the contextual, perceptual and affective features. Travelling mentally back into one’s personal past is referred to as *episodic memory*, while retrieving specific features belonging to these episodic memories is a cognitive capacity involving *source-memory* processes. Source-memory is embedded into the episodic memory, and triggers semantic processes aiming at retrieving the origin of a memory and enabling to distinguish between two or more episodic memories. In humans, source-memory is mostly studied using item *versus* source-memory discrimination task. As the memory of the source relies on the recall of specific characteristics of a prior situation, participants are asked to retrieve the features of the context in which items were previously encountered. In such studies, participants have to recall the items they encountered earlier in opposition to new items (item memory), and then retrieve the context in which they were presented (*e.g*. whether the target items were read or mentally imagined^2^; their spatial location^3^; the list to which they belonged^4^; the colour of the item^5^, etc; source-memory). Only few studies have focussed on source-memory in non-human animals. One single experiment mimicked the item *versus* source procedure in monkeys^6^. Rhesus monkeys learnt to respond differently to two images (*i.e*. the first needed to be simply touched and the second one should be classified as bird, fish, flower, or human). At test, four images were presented (the two previously seen images and two distractors) and half of the monkeys needed to retrieve the image previously simply touched, and half of the monkeys needed to retrieve the image previously classified. Monkeys showed their ability to discriminate between the two sources when test was presented after a short delay, but they made source-memory mistakes when tested after a long delay, while still avoiding distractors (item memory preserved). Crystal and colleagues^7^ studied rat’s ability to discriminate between self- or externally-generated information. This study focused on another type of source-memory, called reality monitoring (i.e. did I learn this information myself, or did I learn it from someone else?^1^). Apart from monkeys and rats, source-memory has not been investigated in other species, and it is not known to what degree the ability to remember the source of an event is a shared capacity between species or if it is a specific cognitive feature of mammals.

Cuttlefish (*Sepia officinalis*) belong to the group of coleoid cephalopod molluscs. They are active predators, using both visual and olfactory cues for defensive behaviours, foraging, and inter-individual communication. Cuttlefish mostly rely on visual information to camouflage in their surroundings to hide from prey and avoid predators^8^, but chemical perception is also a crucial sense for the detection of both prey and predators^9–11^. They can communicate with conspecifics visually^11^, and select the most sexually available partner based on chemical cues^12,13^. Cuttlefish possess a centralized nervous system, and exhibit advanced cognitive abilities which have arisen independently of the vertebrates. By remembering what they have eaten, and where and how long ago they ate, cuttlefish are able to adapt their foraging behaviour according to the time of replenishment of different types of prey^14^. This study provided the first evidence of episodic-like memory in an invertebrate.

The capacity to retrieve an episodic memory is based at first on the quality of the encoding, depending on perception and sensitivity. Perception is an essential capacity for an organism to adapt its environment. It sorts appropriate information coming from the senses, to build adapted representations of the environment. This capacity to retrieve what was perceived depends on personal assessment of past internal sensations. Where episodic-like memory is based on external information (i.e. what-where-when), investigating the source *via* the retrieval of perceptive signal is based on internal information (e.g. did I smell or did I see) supported by the senses, which provides more information about a possible subjective experience of the animal (“what did I just felt?”). In this study, we assessed cuttlefish ability to discriminate between two different modalities (*i.e*. visual and olfactory; “did I see or did I smell?”) and then, to retrieve which modality was previously encountered. In experiment 1, cuttlefish were trained to discriminate visual and olfactory *stimuli* of a crab, and in experiment 2, cuttlefish were trained to discriminate visual and olfactory *stimuli* of crabs, fish, and shrimp randomly presented. Each experiment was divided into: 1) sessions of training where the cuttlefish were trained to associate a panel with a sensory modality (e.g. panel n°1 associated to olfactory presentation of the item), 2) transfer tests (i.e. novel item) without delay to test whether cuttlefish have learnt the rule see *vs* smell (experiment 2 only), and 3) delay tests, with a delay between presentation of the item and choice between the panels, to assess cuttlefish ability to retrieve whether they smelt or saw an item when unexpectedly asked.

## Results

### Experiment 1

**Figure 1:**
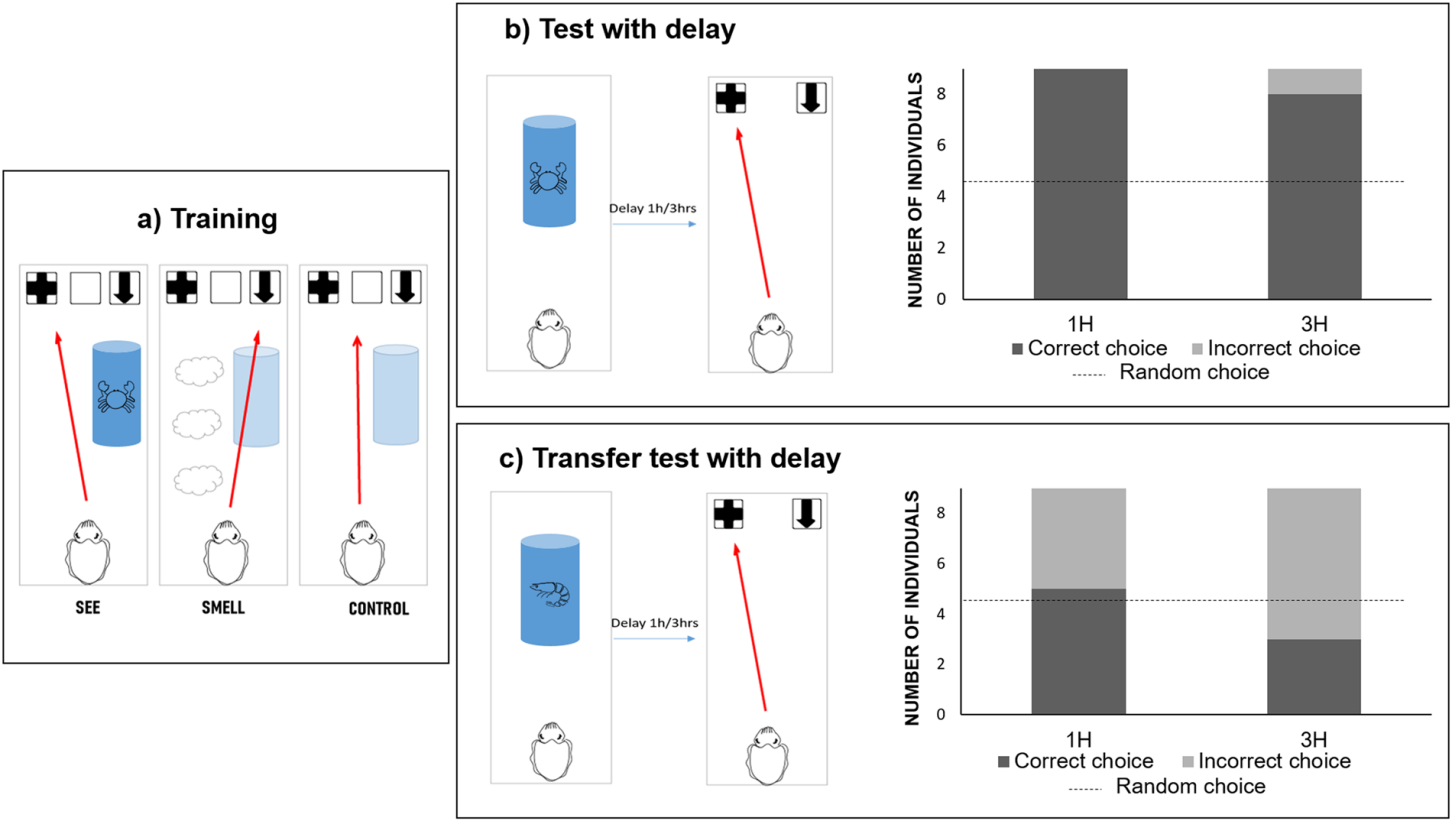
Experimental procedures and results for Experiment 1. (**a**) Training session: cuttlefish were presented with three different experimental conditions. SEE condition where the visual stimulation of a crab was associated with the left panel (*i.e*. panel n°2); SMELL condition where the olfactory stimulation of a crab was associated with the right panel (i.e. panel n°1); CONTROL condition with no presentation of visual and olfactory stimulation, associated with the central panel (*i.e*. panel n°3). (**b**) Delay test: cuttlefish were presented with visual or olfactory stimulation of a crab. After a delay, they had the opportunity to make a choice between panel n°1 and panel n°2. All the cuttlefish chose the correct panel after 1 h delay, and the majority of cuttlefish chose the correct panel after 3 hrs delay. (**c**) Delay transfer test: cuttlefish were presented with visual or olfactory stimulation of a shrimp. After a delay they had the opportunity to make a choice between panel n°1 and panel n°2. 5 cuttlefish passed the transfer test after 1 h delay, and 3 cuttlefish passed the transfer test after 3 hrs delay.

Cuttlefish were first trained to associate panels with different graphic patterns with the modality of presentation of a crab (Fig. 1a). Cuttlefish were randomly tested in three different experimental conditions: *visual condition* (crab presented inside a glass tube), *olfactory condition* (crab odour poured in the tank), and *control condition* (no visual or olfactory *stimuli* added at the beginning of the trial). Training ended when cuttlefish chose the correct panel according to the experimental condition at least 8 times out of 10 consecutive trials (Binomial test with 1/3 probability of success: *p* = 0.003, confidence interval: 0.44–0.97). Cuttlefish required an average of 56 trials to reach the acquisition criterion. Following training, a delay test was undertaken: a crab was presented using either olfactory or visual cues. After a delay, only panels associated to visual and olfactory conditions were placed in the tank to test cuttlefish ability to retrieve whether they smelt or they saw the crab before (Fig. 1b; for details see Methods). Results showed that all cuttlefish chose the correct panel according to the modality of presentation of the crab encountered 1 h before (Binomial test: *p* = 0.0039, confidence interval: 0.66–1). Cuttlefish were tested the following day with a 3 h delay, and most of them correctly retrieved the modality of presentation of the crab encountered before (*p* = 0.039, confidence interval: 0.52–0.99). However, during a delay transfer test (novel item; Fig. 1c), cuttlefish were not able to retrieve the panel corresponding to the presentation of a novel prey after 1-hour and 3-hours delays (Binomial test: 1-hour delay, *p* = 1, confidence interval: 0.21–0.86; 3-hours delay, *p* = 0.50, confidence interval: 0.075–0.70).

### Experiment 2

**Figure 2:**
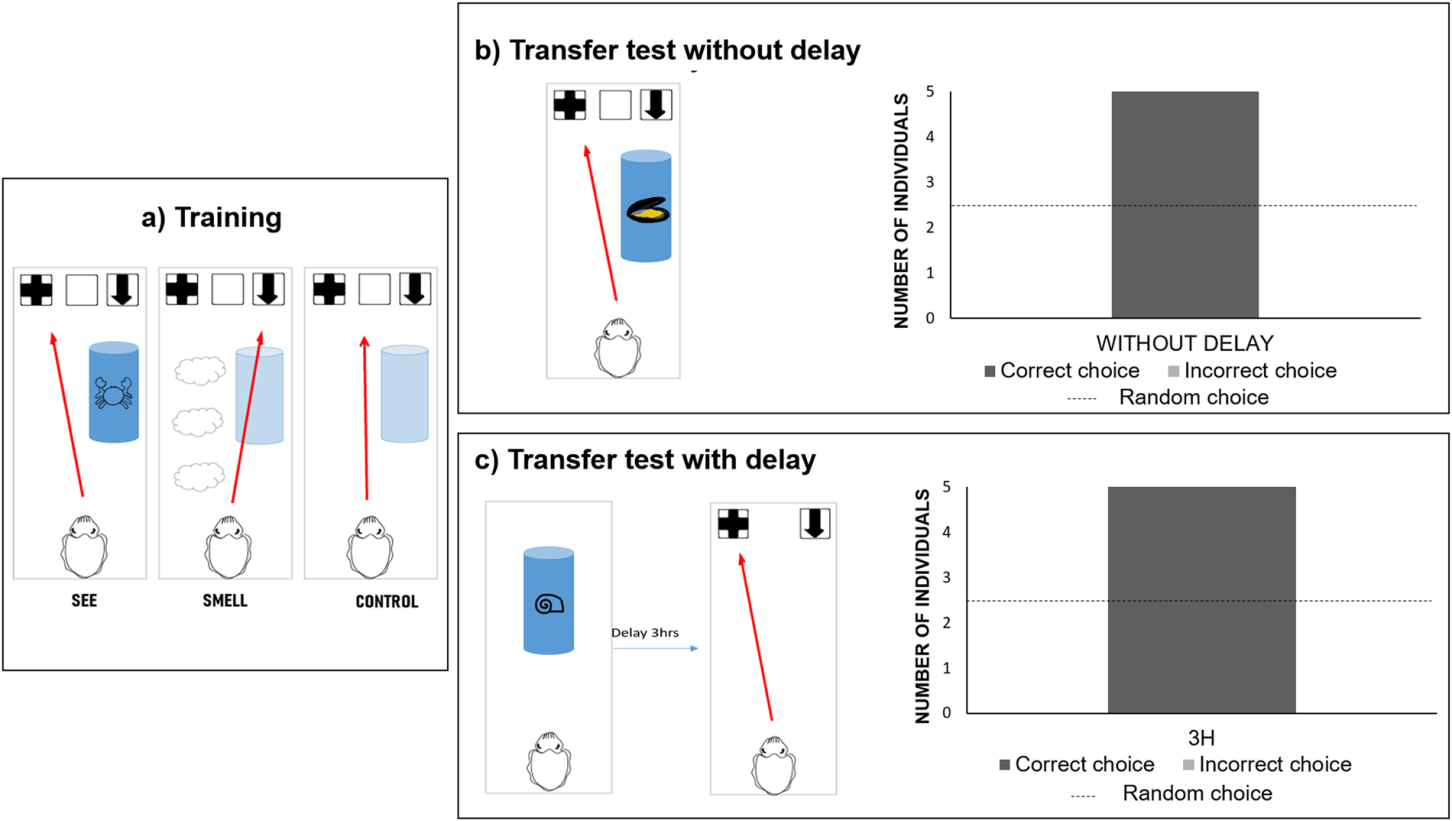
Experimental procedures and results for Experiment 2. (**a**) Training session: the experimental set-up was identical to the first experiment, except that cuttlefish were randomly presented with visual and olfactory stimulations of fish, crabs, and shrimp. (**b**) Transfer tests without delay: cuttlefish were presented with visual and olfactory stimulation a novel item never encountered before. All the cuttlefish managed to pass this transfer test without delay. (**c**) Delay transfer test: cuttlefish were presented with a novel item. After 3 hrs delay, cuttlefish had the opportunity to make a choice between panel n°1 and panel n°2. All the cuttlefish passed the transfer test with delay.

In experiment 2, cuttlefish were trained with three different items (*i.e*. fish, crab, and shrimp, Fig. 2a). All cuttlefish reached the learning criterion (Binomial test with 1/3 probability of success: *p* = 0.003, confidence interval: 0.44–0.97). Once cuttlefish reached the learning criterion, transfer tests were realized (without delay between presentation of the item and choice of a panel; Fig. 2b). Once cuttlefish succeeded these transfer tests, they were tested with a 3 h delay (i.e. delay transfer test; for details see Methods; Fig. 2c). All the cuttlefish (five out of five) tested were able to retrieve the correct panel linked with the presentation of the new items after 3-hours delay.

## Discussion

We showed that cuttlefish are able to learn a discrimination rule based on two sensorial modalities (vision *versus* olfaction) and are able to retrieve the modality of presentation of an item presented before a long-term delay.

In Experiment 1, cuttlefish were able to retrieve the modality of presentation of a crab encountered 1-hour and 3-hours before. However, they were not able to perform the task with a novel prey (i.e. shrimp). This result suggests that cuttlefish likely used an associative-learning based strategy to perform the task, associating the crab’s characteristics (i.e. odour of crab, sight of crab) with the visual cues, instead of generalizing the discrimination rule “see *vs* smell”. To facilitate learning of this general rule, we ran Experiment 2 using several items during training (i.e. crabs, shrimps, and fish). We ran a transfer test without delay after training to establish whether the cuttlefish were able to generalize the rule to new items. In this second experiment, the cuttlefish were able to generalize their discrimination learning to new items, showing that they extracted the rule “see *versus* smell”. This last result shows that cuttlefish did not use lower-level cognitive processes to solve the task as they did in the first experiment.

To our knowledge, our study is the first to demonstrate the ability to discriminate between two sensory modalities in animals. Under the visual modality, several studies showed animals’ capacity to discriminate between colours (e.g. bees^15^; chimpanzees^16^; weanling pigs^17^; seals^18^; horses^19^; cats^20^; ravens^21^), shapes or positions (e.g. mice^22^; octopus^23^), and movements (e.g. pigeons^24^). The ability to discriminate between olfactory cues was also shown in several species using odours and flavours (e.g. rats^25,26^; bees^27^; dogs^28^; bats^29^; pandas^30^; pigs^31^). Other studies used multimodal cues to evaluate their interaction or facilitation effect on a discrimination task. For instance, Verbaal and Luksch^32^ investigated the effect of audiovisual *stimuli* in comparison to audiory and visual *stimuli* alone on discrimination capacities in chicken. Studies have yet to investigate the animal’s capacity to discriminate the sensation *per se* (i.e. do you *see* or do you *smell*?). Previous studies focused on one sensorial modality at a time (e.g. visual *or* olfactory), or used multimodal cues for other purposes. Our study also brings new insights on cephalopods’ discrimination abilities. It has been shown that cuttlefish can visually discriminate brightness, substrate texture^8^, graphic patterns^33,34^, and olfactory discriminate odours from congeners, food or predators^9,35^. But it is the first time that a study shows cuttlefish ability to recognize and discriminate between olfactory and visual *stimuli*.

In item *versus* source tasks used to investigate source-memory in humans, participants are not aware that they will be asked about the source of information at the time of encoding. In our study, cuttlefish were trained to repeatedly choose the correct panel according to the modality of presentation of an item (*i.e*. visual or olfactory modalities). This experimental design induced learning of a semantic rule: *i.e*. association sensory modalities/panels. At test, using a new item and adding a delay allowed us to unexpectedly ask cuttlefish whether they smelt or saw the item before. This procedure was designed to avoid cuttlefish to explicitly encode the sensory modality at the time of presentation of the item, as cuttlefish was not aware that it will be asked to answer the question see *vs* smell later. Nevertheless, it is possible that cuttlefish chose by familiarity the correct panel according to the last modality (*i.e*. olfactory or visual) sensed when the item was presented. In further studies, the effect of familiarity will be countered by successively exposing cuttlefish to two items (one visually and the other olfactory) in different contexts. At test, cuttlefish will be asked to retrieve the modality of presentation of the first or second item according to the context, to control sensory memory trace^36^.

Our study provides the first evidence that cuttlefish are able to discriminate and retrieve their own visual and olfactory sensations. This finding is a real advance in the study of episodic cognition in animals. The classical debate as to whether mental time travel is unique to humans opposes (1) the capacity of subjective experience observed in humans, (2) to the capacity of “simply” form sequential mnemonic representations of personal past episodes^37^. In classical episodic-like memory tasks, subjective experience is not assessed as animals need to retrieve external information only (e.g. what-where-when^38^). In absence of language, it is still impossible to investigate subjective experience in animals. However, our design might be useful to approach the question of subjectivity as it is based on animals’ capacity to retrieve an internal information from a previous event (*i.e*. own perception). The source-monitoring framework specifies that an episodic memory is retrieved when *several* signals are brought back in mind. In our experiment, cuttlefish were trained to retrieve a single perceptive signal. In future studies, it would be necessary to test whether cuttlefish are able to remember different signals in an integrate representation, such as for instance a perceptive and a contextual signal.

## Methods

### Ethical statement

Experiments were carried out in accordance with directive 2010/63/EU (European parliament) and the French regulation relative to the protection and use of animals in research. Procedures were approved (#22429 2019101417389263 v2) by the regional ethical committee (Comité d’Ethique Normandie en Matière d’Expérimentation Animale, CENOMEXA; agreement number 54).

### Subjects

The experiments were carried out in sub-adult European common cuttlefish (*Sepia officinalis*) ranging in age from 3 to 6 months at the start of experiment 1 (N = 9) and from 9 to 12 months at the start of experiment 2 (N = 6). Five out of the six cuttlefish tested in experiment 2 were reused from experiment 1. Cuttlefish were reared from eggs collected in the English Channel, at the CREC (Centre de Recherches en Environnement Côtier – Marine Station of the University of Caen, Luc-sur-Mer, France). Cuttlefish were housed individually in grey plastic tanks (80 × 60 × 40 cm) with natural circulating seawater (temperature: 15 ± 1 °C). Cuttlefish were maintained under artificial light conditions (12L:12D cycle) and were fed daily with live crabs (*Carcinus manenas*) and shrimp (*Crangon crangon*) of suitable size before starting the experiments. One cuttlefish was removed from the experiment before the final test because it started to display an unusual swimming behaviour.

### Experimental conditions

The panels used in the experiments consisted of 10 cm white plastic squares with or without a black geometric shape in the middle: panel n°1 pictured a graphic arrow, panel n°2 pictured a cross, and panel n°3 was devoid of drawing. Cuttlefish were trained to associate each panel with different experimental conditions:

- SMELL condition: Association panel n°1/olfactory stimulation. Cuttlefish were trained to go close to the panel n°1 when seawater with a *stimulus* odour was poured in the tank (*i.e*. experiment 1: odour of crab; experiment 2: odour of crab, fish or shrimp). To prepare olfactory stimulus for SMELL conditions, items were placed in a bucket with seawater so that their smell spread in the water (about one liter of water for one adult crab (*Carcinus maenas*), three liters for one adult fish (*Dicentrarchus labrax*), and one liter for five adult shrimp (*Crangon crangon*)).
- SEE condition: Association panel n°2/visual *stimulus*. Cuttlefish were trained to go close to the panel n°2 when a visual *stimulus* was presented inside the glass tube (*i.e*. experiment 1: one live crab; experiment 2: one live crab, juvenile fish or shrimp).
- CONTROL condition: Association panel n°3/absence of additional olfactory or visual stimulation. Cuttlefish were trained to go close to the panel n°3 when no additional visual *stimulus* (*i.e*. glass tube empty) or olfactory *stimulus* (*i.e*. natural seawater without *stimulus* odour poured in the tank) was presented. Control condition is necessary to make cuttlefish learn the association between panel n°1/olfactory stimulation, and not panel n°1/nothing in the glass tube.

### Procedure

#### Experiments 1&2: Pre-training: learning to approach a panel to get food

##### Step 1: Familiarization.

To familiarize cuttlefish with the presence of panels inside their tank, the panel n°3 was placed in their home tank during each feeding session during a week.

##### Step 2: Approaching a panel to get a food reward.

The panel n°3 was placed in the tank; after 60 seconds, a prey was placed just in front of it. If the cuttlefish did not catch the prey within 3 min, both the panel and the prey were removed from the tank. This procedure was repeated four times a day. When a cuttlefish went repeatedly close (*i.e*., less than 10 cm) to the panel before placing the prey in the tank, cuttlefish started training. We considered that cuttlefish had learned the task when they went close to the panel in less than 60 seconds after it was placed inside their home tank, at least 8 times in 10 consecutive trials.

#### Experiments 1&2: Training: learning to approach a distinct panel according to the experimental condition

Cuttlefish were tested four times a day. Each experimental condition was presented in randomized order (StatTrek.com). At the beginning of a trial, whatever the experimental condition, the same gestures were repeated by the experimenter: (1) a glass tube (higher than the water surface) with or without visual *stimulus* inside was gently placed inside the tank, (2) a 500 mL beaker full of natural seawater with or without additional olfactory *stimulus* was carefully poured in the tank, and (3) the three panels were placed along one of the walls of the tank. Cuttlefish were rewarded (one shrimp or one crab) when they came, closer than 10 cm, in front of the correct visual cue. When the prey was caught by the cuttlefish, the glass tube and the panels were removed from the tank. When the first panel approached by cuttlefish was the correct one according to the experimental condition, this was considered as a correct choice. When cuttlefish failed to go close to the correct panel within 4 minutes, the three panels and the glass tube were removed from the tank. When cuttlefish approached the correct cue but not at first or did not approached it within 4 minutes, this was considered as incorrect choice. Cuttlefish were trained until they reached a learning criterion established as eight correct choices out of ten consecutive trials (Binomial test: probability to choose the correct visual cue: probability of success = 1/3; *p* = 0.003).

#### Experiment 1: Test phase

To make sure cuttlefish choose a panel according to the kind of stimulation encountered before the delay and not currently experiencing, only panels n°1 (SMELL) and n°2 (SEE) were introduced in the tank at tests and transfer tests with delay.

##### Delay test: Have you seen or smelt a crab before?

When the learning criterion was reached, the test phase began. For the first test trials, a delay was introduced between the presentation of the *stimulus* (*i.e*. crab odour poured in the tank or crab placed inside the glass tube) and the introduction of the panels inside the tank. Panels n°1 (SMELL) and n°2 (SEE) were introduced in the tank. Each cuttlefish was tested once with two different delays (1 hour and 3 hours), one test was performed per day (day 1: 1 hour; day 2: 3 hours delay), with some trials were ran in-between. The experimental conditions (SMELL or SEE) used were randomly chosen.

##### Delay transfer tests: Have you seen or smelt a shrimp before?

The procedure described before was repeated using a shrimp as a *stimulus* instead of a crab. Each cuttlefish was tested once with two different delays (1 hour and 3 hours), the experimental conditions (SMELL or SEE) used being randomly chosen.

#### Experiment 2: Test phase

##### Transfer tests without delay: Do you see or smell?

When the learning criterion was reached, cuttlefish were first presented with a transfer test once to check whether they actually learnt to distinguish SMELL *versus* SEE conditions. In this test, the procedure used was the same than during training repeated *once* with a novel *stimulus* (*e.g*. mussel, snail, seaweed, etc.). If the transfer test was successful (*i.e*. the cuttlefish choose the panel associated with the modality of presentation of the prey) cuttlefish went to the next step (*i.e*. delay transfer test). If the transfer without delay was not successful, cuttlefish went back to training until they reached the learning criterion another time, and another transfer test without delay was realized (with a novel prey each time).

##### Delay transfer tests: Have you seen or smelt an item before?

Once cuttlefish successfully passed the transfer test without delay, they were tested with a delay. The procedure used was the same as described in “Delay transfer test” for experiment 1, except: that the *stimulus* presented was neither used for training nor transfer tests without delay, and that only one delay interval was used: three hours.

### Statistical analyses

All data were analysed with non-parametric tests and computed using R software (version 3.5.1). We determined a learning criterion of 8 correct answers out of 10 consecutive trials with a probability of success of 1/3. To analyse whether most cuttlefish significantly chose the correct panel after the delay, we used exact binomial tests (binom.test function on R). *P* values and confidence intervals were reported.

## Data Availability

Data are available on datadryad.org: 10.5061/dryad.h44j0zpfx.
